# The Diagnostic Yield of Video Capsule Endoscopy for Uncomplicated Asymptomatic Iron Deficiency Anemia With Negative Bidirectional Endoscopy in a Large Military Gastroenterology Program

**DOI:** 10.7759/cureus.81356

**Published:** 2025-03-28

**Authors:** Carl Kay, Tyler H Doty, Cassandra L Craig, Samuel C Owen

**Affiliations:** 1 Department of Gastroenterology, Brooke Army Medical Center, San Antonio, USA; 2 Department of Internal Medicine, Brooke Army Medical Center, San Antonio, USA

**Keywords:** capsule endoscopy, diagnostic yield, iron-deficiency anemia, retrospective study, small bowel bleeding

## Abstract

Background: Iron-deficiency anemia (IDA) often results from gastrointestinal (GI) bleeding. Video capsule endoscopy (VCE) is increasingly used to investigate suspected small bowel bleeding, particularly when bidirectional endoscopy is nondiagnostic. However, the diagnostic yield of VCE in asymptomatic patients with IDA and negative bidirectional endoscopy is not well-established, leading to variability in clinical guidelines.

Methods: We conducted a retrospective review of patients at Brooke Army Medical Center who underwent VCE from January 2019 to April 2023 following negative bidirectional endoscopy for IDA, defined as ferritin <45 ng/mL. We analyzed demographic data, VCE findings, and outcomes, including need for further intervention, persistence of IDA, and hospitalizations. We also assessed the impact of referral delays on outcomes.

Results: Out of 238 patients, 75 met the inclusion criteria. VCE revealed abnormal findings in 36% of cases, with 8% having actionable findings necessitating repeat endoscopy. The most common abnormality was small bowel angiodysplasias. No small bowel malignancies were detected. Patients aged over 65 had a higher rate of abnormal findings and actionable lesions. Delays in subspecialty evaluation (>180 days) did not significantly affect patient outcomes.

Conclusion: In asymptomatic IDA patients with negative bidirectional endoscopy, VCE demonstrates a 36% diagnostic yield, primarily identifying small bowel angiodysplasias, with limited impact on detecting malignancies. This study highlights the importance of individualizing VCE use to each patient, and suggests that delays in referral do not adversely affect outcomes. Further prospective studies are needed to refine diagnostic guidelines and enhance cost-effectiveness.

## Introduction

Iron-deficiency anemia (IDA) is a common condition in the United States, requiring extensive diagnostic evaluations that are both time-consuming and contribute significantly to the economic burden on our healthcare system [1,2]. Gastrointestinal (GI) bleeding is one of the most common underlying causes of IDA [3]. Video capsule endoscopy (VCE) has emerged as the primary modality tool for investigating suspected small bowel bleeding, particularly in cases where traditional bidirectional endoscopy, encompassing esophagogastroduodenoscopy (EGD) and colonoscopy, fails to identify a source of bleeding [4].

Most studies aimed at identifying the diagnostic yield of VCE have focused on symptomatic patients, so it is inappropriate to apply it to patients with negative bidirectional endoscopy and no overt GI bleeding [5-16]. Significant heterogeneity in studies confounds systematic reviews and meta-analyses [17]. This discrepancy has resulted in disparate society guidelines regarding the use of VCE [18-20].

To address this knowledge gap, our study aimed to evaluate the diagnostic yield of VCE specifically in asymptomatic patients with IDA (rigorously defined as ferritin <45 ng/mL) who have negative bidirectional endoscopy [21]. Furthermore, we closely evaluated age and antithrombotic use as potential risk factors. Additionally, we assessed the impact of referral delays and the timing of VCE evaluation on patient outcomes. He hypothesized that VCE would have a modest diagnostic yield in this patient population, with a low yield of actionable findings.

## Materials and methods

A retrospective review was performed on patients who underwent VCE after a nondiagnostic bidirectional endoscopic exam to evaluate IDA at Brooke Army Medical Center from January 2019 through April 2023, with Institutional Review Board approval and conducted in accordance with ethical guidelines. The study population included active-duty military service members, dependents, and military retirees with laboratory-confirmed IDA (hemoglobin <13 g/dL in men and <12 g/dL in nonpregnant women with concurrent serum ferritin <45 ng/mL). Exclusion criteria included overt GI bleeding symptoms, history of inflammatory bowel disease, history of myocardial infarction/stroke/severe infection/respiratory failure within six weeks of obtaining VCE, pregnant patients, age less than 18 or older than 89 years old, history of small bowel obstruction, history of esophageal disease or upper gastroenterological surgery that precluded endoscopic passage of the capsule, known GI or hematologic malignancy, or a known alternative source of blood loss (including but not limited to menstrual bleeding).

Data on patient age, sex, race, ethnicity, smoking status, comorbidities (including coronary artery disease, chronic kidney disease, and diabetes), medications (antiplatelets, anticoagulation, and iron supplementation), and laboratory studies (baseline hemoglobin, ferritin, and mean corpuscular volumes, MCVs) were collected. The date of the patient’s initial referral to gastroenterology for IDA, date of initial endoscopies, and date of VCE were also obtained to determine the number of days from diagnosis until completion of evaluation. Manual chart review of patient records was performed to identify key patient outcomes, including the necessity and quantity of blood transfusions, the number of hospitalizations, persistence of IDA at six months from initial diagnosis, the requirement of intravenous iron repletion, and all-cause mortality.

All VCE procedures were performed with the same patient instructions between January 2019 and April 2023. Patients were instructed to cease oral iron medications three days before VCE and have only a clear liquid diet the day before the procedure. The evening before the procedure, patients were instructed to consume 1 L of low-volume polyethylene glycol lavage solution with 16 ounces of clear liquids. On the morning of the procedure, patients were instructed to fast and take 2.4 mL of simethicone orally 30 minutes before the procedure. After ingesting the video capsule, patients were instructed to fast for two more hours. Morning medications and clear liquids were permitted two hours after ingesting the video capsule. Four hours after ingesting the video capsule, a light snack was permitted. Eight hours after ingesting the video capsule, normal diet was resumed.

All VCE reports were reviewed by a single, board-certified staff gastroenterologist. Findings were categorized as follows: normal small bowel mucosa, ulcer, erosion/inflammation, vascular lesion, nonbleeding polyp/mass, other (whipworms), gastropathy, duodenopathy (within the proximal duodenum), and colopathy. Abnormal VCE findings were defined as any VCE that was not described as normal small bowel mucosa. Abnormal VCE findings were further subcategorized as actionable VCE findings if the suspected culprit lesion was identified or if VCE findings prompted subsequent intervention (e.g., repeat endoscopy, therapeutic endoscopic procedure, and/or change in management). Additional data collection included completeness of the exam (e.g., equipment failure, retention in the stomach, or failure to reach the cecum), need for a repeat VCE, and whether the finding(s) prompted a repeat endoscopy. We also examined any hospitalizations attributable to capsule findings, need for blood transfusion, or additional procedures (e.g., repeat EGD, push enteroscopy, repeat colonoscopy). Although all VCEs were performed at a single center, hospitalizations, radiographs, and laboratory findings could be identified across military and affiliated facilities through the shared electronic medical record.

JMP software version 13.2 (SAS Corp., Cary, NC) was used for data analyses. Normally distributed continuous variables were reported as mean ± standard deviation. Median and interquartile range (IQR) were calculated for nonnormally distributed continuous variables. Categorical variables were reported as counts and percentages. Two-sided t-test and analysis of variance were used to compare the means of continuous variables. The Kruskal-Wallis test was used to compare medians for variables not normally distributed. The Chi-square test was used to compare categorical variables.

## Results

**Figure 1:**
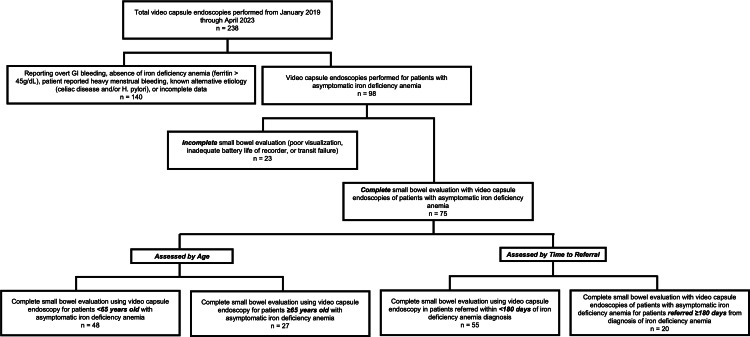
Flow diagram of patients This flowchart depicts the selection and classification of patients who underwent VCE for asymptomatic IDA between January 2019 and April 2023. After identifying appropriate VCE studies and excluding incomplete small bowel evaluations, patients were analyzed based on age and time to referral. The subdivisions for age (<65 vs. ≥65 years) and referral timing (<180 vs. ≥180 days from IDA diagnosis) are not mutually exclusive, meaning a single patient could be included in both categories if they met the respective criteria VCE: video capsule endoscopy; IDA: iron-deficiency anemia; GI: gastrointestinal

At our facility, a total of 238 patients underwent VCE between the years 2019 and 2023. Excluded from the study were 140 patients (58.8%) due to reporting overt GI bleeding, absence of IDA (ferritin >45 ng/dL), alternative etiology of bleeding identified (e.g., menstrual bleeding), alternative etiology of IDA (celiac disease and/or *Helicobacter pylori* infection), or incomplete data. The final analysis was completed on 75 patients (Figure 1). The mean age of the patients was 58.7 ± 16.9, with 37 (37.3%) being female and 62 (69.3%) being White patients. Baseline hemoglobin average was 10.3 ± 2.2 g/dL, ferritin of 19.8 ± 13.1 ng/mL, and MCV of 81.6 ± 10.9 fL. Of the 75 VCE studies that met criteria, 27 (36%) had abnormal findings (ulcer, erosion/inflammation, vascular lesion, nonbleeding polyp/mass, other/whipworms, gastropathy, duodenopathy, and colopathy), but only six (8%) had actionable findings requiring a repeat endoscopy with planned intervention (Table 1). The most prevalent abnormal VCE finding was small bowel angiodysplasias (13 patients; 17.3%). Only one (1.3%) of these patients had active bleeding identified from this source on repeat endoscopic evaluation. There were no findings of small bowel malignancies on VCE. The median follow-up time from the date of VCE was 3.1 years, with an IQR of 2.3-3.7 years.

**Table 1:** Overall patient demographics, VCE findings, and outcomes VCE: video capsule endoscopy; IDA: iron-deficiency anemia; SD: standard deviation; BMI: body mass index; IV: intravenous; MCV: mean corpuscular volume

Overall parameters (n = 75)	Values
Demographics	
Age, mean ± SD	58.7 ± 16.9
Female sex, n (%)	29 (37.3%)
White race, n (%)	52 (69.3%)
African American race, n (%)	11 (14.6%)
Hispanic ethnicity, n (%)	5 (6.7%)
Past medical history	
Any tobacco use, n (%)	25 (33.3%)
BMI, mean ± SD	29.9 ± 5.6
Coronary artery disease, n (%)	12 (16%)
Chronic kidney disease, n (%)	6 (8%)
Diabetes, n (%)	22 (29.3%)
Medication use	
Aspirin use, n (%)	21 (28%)
Any antiplatelet use, n (%)	21 (28%)
Oral anticoagulation use, n (%)	12 (16%)
Labs	
Baseline hemoglobin (g/dL), mean ± SD	10.3 ± 2.2
Baseline MCV (fL), mean ± SD	81.6 ± 10.9
Baseline ferritin (ng/mL), mean ± SD	19.8 ± 13.1
Time of evaluation	
Time to IDA referral, days, mean ± SD	161 ± 254
Time to bidirectional endoscopy, days, mean ± SD	220 ± 282
Time to VCE, days, mean ± SD	349 ± 355
VCE findings	
Abnormal VCE finding, n (%)	27 (36%)
Actionable VCE finding, n (%)	6 (8%)
Normal VCE, n (%)	48 (64%)
Erosion/inflammation on VCE	6 (8%)
Vascular lesion on VCE	13 (17.3%)
Nonbleeding polyp/mass	2 (2.7%)
Repeat VCE required	0 (0%)
VCE findings were within the reach of standard bidirectional endoscopy	6 (8%)
Outcomes	
Required transfusion, n (%)	19 (25.3%)
Required hospitalization, n (%)	19 (25.3%)
Persistent IDA at six months, n (%)	38 (50.7%)
Required IV iron, n (%)	19 (25.3%)
All-cause mortality at 12 months, n (%)	0 (0%)

There were 27 patients (36%) in our study who were over 65 years old. This older population had higher rates of comorbidities, including coronary artery disease (n = 8, 29.6% vs. n = 4, 8.3%; p = 0.016) and chronic kidney disease (n = 5, 18.5% vs. n = 1, 2%; p = 0.012) (Table 2). Rates of aspirin, antiplatelets, and anticoagulants were also higher in this population. They had significantly higher rates of abnormal findings on VCE (n = 16, 59.9% vs. n = 11, 22.9%; p = 0.002), actionable findings (n = 6, 22.2% vs. n = 0, 0%; p <0.001), and need for subsequent endoscopy (n = 7, 25.9% vs. n = 0, 0%; p <0.001) (Table 2).

**Table 2:** Baseline patient demographics, VCE findings, and outcomes based on age Chi-square tests were used to assess associations between age groups and variables, with corresponding chi-square values and p values reported to indicate statistical significance (p < 0.05 is considered significant) VCE: video capsule endoscopy; IDA: iron-deficiency anemia; SD: standard deviation; BMI: body mass index; IV: intravenous; MCV: mean corpuscular volume

Parameters	Age ≥ 65 years old (n = 27)	Age <65 years old (n = 48)	Chi-square value	p value
Demographics				
Female sex, n (%)	9 (33.3%)	19 (39.6%)	0.289	0.591
White race, n (%)	19 (70.3%)	33 (68.8%)	4.178	0.884
African American race, n (%)	4 (14.1%)	7 (14.6%)	4.178	0.978
Hispanic ethnicity, n (%)	2 (7.4%)	3 (6.3%)	0.599	0.847
Past medical history				
Any tobacco use, n (%)	13 (48.1%)	13 (25%)	4.519	0.066
BMI, mean ± SD	30.5 ± 5.2	30 ± 5.7	0.362	0.542
Coronary artery disease, n (%)	8 (29.6%)	4 (8.3%)	5.831	0.016
Chronic kidney disease, n (%)	5 (18.5%)	1 (2%)	6.342	0.012
Diabetes, n (%)	11 (40.7%)	11 (22.9%)	2.648	0.104
Medication use				
Aspirin use, n (%)	12 (44.4%)	9 (18.7%)	5.659	0.017
Any antiplatelet use, n (%)	12 (44.4%)	9 (18.7%)	5.659	0.017
Oral anticoagulation use, n (%)	11 (40.7%)	2 (4.2%)	16.132	<0.001
Labs				
Baseline hemoglobin (g/dL), mean ± SD	10.2 ± 2	10.7 ± 2.5	1.281	0.264
Baseline MCV (fL), mean ± SD	84.9 ± 7.7	78.7 ± 13.1	5.451	0.023
Baseline ferritin (ng/mL), mean ± SD	19.3 ± 9.9	18.1 ± 13.3	0.551	0.458
Time of evaluation				
Time to IDA referral, days, mean ± SD	145 ± 215	119 ± 174	0.163	0.693
Time to bidirectional endoscopy, days, mean ± SD	192 ± 231	195 ± 195	0.022	0.885
Time to VCE, days, mean ± SD	305 ± 297	353 ± 379	0.073	0.782
VCE findings				
Abnormal VCE finding, n (%)	16 (59.3%)	11 (22.9%)	22.139	0.002
Actionable VCE finding, n (%)	6 (22.2%)	0 (0%)	13.725	<0.001
Normal VCE, n (%)	11 (40.7%)	37 (77.1%)	9.524	0.002
Outcomes				
Required subsequent endoscopic intervention, n (%)	7 (25.9%)	0 (0%)	9.524	<0.001
Required transfusion, n (%)	7 (25.9%)	9 (18.8%)	0.770	0.467
Required hospitalization, n (%)	10 (37.1%)	9 (18.8%)	3.055	0.080
Persistent IDA at six months, n (%)	17 (63.0%)	21 (43.8%)	2.552	0.110
Required IV iron, n (%)	4 (14.8%)	6 (12.5%)	0.351	0.777
All-cause mortality at 12 months, n (%)	0 (0%)	0 (0%)	0	-

Patients who had delayed time to subspecialty evaluation, defined in our study as >180 days from the diagnosis of IDA to evaluation by gastroenterology, had no significant difference in outcomes (Table 3). While there was a trend to more hospitalizations in the early referral cohort, there were no significant differences in abnormal VCE findings, actionable findings, transfusion requirements, persistence of IDA, or all-cause mortality.

**Table 3:** Baseline patient demographics, VCE findings, and outcomes based on time to referral Chi-square tests were used to assess associations between age groups and variables, with corresponding chi-square values and p values reported to indicate statistical significance (p < 0.05 considered significant) VCE: video capsule endoscopy; IDA: iron-deficiency anemia; SD, standard deviation; BMI: body mass index; IV: intravenous; MCV: mean corpuscular volume

Parameters	Time to IDA referral ≥180 days (n = 20)	Time to IDA referral <180 days (n = 55)	Chi-square value	p value
Demographics				
Age, mean ± SD	55.9 ± 15.4	58.4 ± 17.6	0.285	0.594
Female sex, n (%)	10 (50%)	18 (32.7%)	1.870	0.171
White race, n (%)	13 (65%)	39 (70.9%)	4.173	0.624
African American race, n (%)	2 (10%)	9 (16.4%)	4.173	0.491
Hispanic ethnicity, n (%)	3(15%)	4 (7.3%)	0.504	0.309
Past medical history				
Any tobacco use, n (%)	5 (25%)	20 (36.3%)	1.218	0.356
BMI, mean ± SD	29.6 ± 4	30.4 ± 6	0.109	0.742
Coronary artery disease, n (%)	4 (20%)	8 (14.5%)	0.325	0.569
Chronic kidney disease, n (%)	0 (0%)	6 (10.9%)	2.372	0.124
Diabetes, n (%)	10 (50%)	12 (21.8%)	5.619	0.018
Medication use				
Aspirin use, n (%)	8 (40%)	13 (23.6%)	1.948	0.163
Any antiplatelet use, n (%)	9 (45%)	13 (23.6%)	2.108	0.072
Oral anticoagulation use, n (%)	2 (10%)	11 (20%)	1.024	0.311
Labs				
Baseline hemoglobin (g/dL), mean ± SD	10.9 ± 2.3	10.4 ± 2.3	1.661	0.191
Baseline MCV (fL), mean ± SD	81.2 ± 9.5	80.8 ± 12.6	0.028	0.873
Baseline ferritin (ng/mL), mean ± SD	20.7 ± 12.3	17.8 ± 12.1	1.573	0.214
Time of evaluation				
Time to bidirectional endoscopy, days, mean ± SD	493 ± 247	85.9 ± 13	40.324	<0.001
Time to VCE, days, mean ± SD	697 ± 427	205 ± 198	29.068	<0.001
VCE findings				
Abnormal VCE finding, n (%)	4 (20%)	22 (40%)	5.369	0.108
Actionable VCE finding, n (%)	0 (0%)	6 (10.1%)	2.807	0.124
Normal VCE, n (%)	16 (80%)	33 (60%)	1.948	0.108
Outcomes				
Required transfusion, n (%)	2 (20%)	15 (27.2%)	2.880	0.114
Required hospitalization, n (%)	2 (10%)	17 (30.1%)	3.390	0.066
Persistent IDA at six months, n (%)	10 (70%)	24 (43.6%)	4.078	0.624
Required IV iron, n (%)	2 (10%)	8 (14.5%)	0.721	0.609
All-cause mortality at 12 months, n (%)	0 (0%)	0 (0%)	0	-

## Discussion

The primary purpose of this retrospective study was to determine the diagnostic yield of VCE for evaluating IDA after negative bidirectional endoscopy, while rigorously excluding any overt bleeding. We found the overall diagnostic yield of finding an abnormality on VCE was 36%, with only 8% requiring a repeat endoscopic exam for intervention following a prior negative bidirectional endoscopy. The most prevalent culprit lesions identified were vascular lesions, specifically small bowel angiodysplasias, and only one small bowel angiodysplasia had concurrent stigmata of bleeding (i.e., active bleeding). There were no occult malignancies during small bowel evaluations.

This study emphasized the significantly higher diagnostic yield of VCE in patients over the age of 65 (n = 16, 59.3% vs. n = 11, 22.9%; p = 0.002). This demographic comprised 36% of the study population and had significantly higher rates of both actionable findings on VCE and high-risk lesions requiring intervention. These findings are likely related to higher rates of comorbidities and anticoagulant/antiplatelet use. We also noted that patients who had a delayed time to subspecialty evaluation, defined in our study as >180 days between the diagnosis of IDA and gastroenterological evaluation, had no significant differences in patient-related outcomes. Given the high prevalence (n = 38, 50.7%) of persistent IDA at six months, this emphasizes the importance of iron repletion amidst diagnostic work-up.

Current guidelines on next treatment steps for uncomplicated asymptomatic patients diagnosed with IDA who have negative bidirectional endoscopy recommend a trial of iron supplementation over routine use of VCE [19]. However, this recommendation comes with very low quality of evidence secondary to the absence of prospective studies and extrapolation from studies that address therapeutics but are not primarily investigating the use of VCE for IDA patients [19]. Our study rigorously defined IDA with ferritin <45 ng/mL, and manual chart review excluded any patients with overt GI bleeding. Therein lies the ultimate strength in our study and what it adds to the current knowledge base. We studied only patients with uncomplicated asymptomatic IDA after negative bidirectional endoscopy.

In contrast to other studies examining the impact of age on VCE findings, we found slightly higher diagnostic yield in patients older than 65 years of age, but lower overall need for interventions and lower overall mortality [7,8]. Compared to these two studies, our study was unique in the rigorous exclusion of any overt bleeding symptoms and definition of IDA with ferritin <45 ng/mL, which is consistent with American guideline recommendations [19]. Similar to these two studies, we found that several capsule endoscopy findings were within the reach of standard bidirectional endoscopy, highlighting the importance of second-look endoscopy, especially in patients with persistent IDA. Despite the modest diagnostic yield of VCE to find abnormalities, our study supports previous studies that suggest that there is a low impact on outcomes [11].

Furthermore, there has previously been an emphasis on diagnosing occult small bowel malignancy and other sinister pathologies [7-9,15,17]. Prior retrospective studies have estimated the diagnostic yield of occult malignancies for VCE in younger patients to be as high as 5% [9]. Our study did not identify any small bowel malignancies, suggesting that the diagnostic yield may be lower in asymptomatic patients after rigorous exclusion of overt bleeding. No other retrospective studies have evaluated the impact of referral delays on diagnostic findings. Our study did not demonstrate any worse patient-related outcomes, with a median follow-up time of 3.1 years (IQR, 2.3-3.7). While this is a common clinical scenario, our study does not suggest that this is an area of quality improvement to reduce adverse outcomes.

There are limitations to consider in our study. First, as a retrospective study, there is a risk for information bias secondary to incomplete or inaccurate documentation of patient and procedure characteristics. Second, the limited options for data extraction in our system mandated manual data entry by our team members, increasing the possibility for human error or subjectivity. Third, there was only one experienced, high-volume VCE reviewer. This increases the possibility of systematic diagnostic error without external validation and precludes assessment of interobserver variability. Additionally, blinding was not feasible given the retrospective nature of the review. Fourth, we had many incomplete small bowel evaluations. Early in the study period, there were faulty VCE monitors with low battery life, which led to terminated recordings prior to reaching the cecum. This led to several excluded VCE studies. Fifth, we did not assess cost-effectiveness, which may limit direct applicability to resource-constrained settings. Sixth and finally, the observations in our study may not be generalizable. All patients who completed screening in our centers were United States military service members, military veteran retirees, or their beneficiaries. The active duty and general civilian populations have differences in health status and exposure to risk factors, with a trend of decreased risk for digestive tract cancers in the military population [22].

## Conclusions

This study provides a detailed retrospective analysis of the yield of VCE when evaluating asymptomatic IDA patients with negative bidirectional endoscopy. By conducting an extensive manual chart review, we thoroughly assessed patient outcomes such as persistence of IDA, need for transfusion, and subsequent interventions. Using an updated ferritin threshold of <45 ng/mL for diagnosing IDA, our findings reproduce the modest diagnostic yield of VCE (36%) with limited actionable results (8%), suggesting that routine use of VCE may be unnecessary. Notably, we reproduced the diagnostic yield of VCE in our military GI program, which cares for military service members, beneficiaries, and their dependents, demonstrating similar findings within this unique patient population. However, higher diagnostic yields in patients over 65 indicate the need for selective use, particularly in those with relevant risk factors. We found that delayed time from IDA diagnosis to gastroenterology evaluation did not significantly impact clinical outcomes, reinforcing the importance of prioritizing iron replacement therapy amidst further work-up and management for IDA. While the interpretation of our results aligns with the data, the absence of a control group and potential confounders limits the generalizability of our conclusions. As such, these findings should be considered hypothesis-generating rather than practice-changing, highlighting the need for prospective studies to refine patient selection and improve cost-effectiveness in small bowel evaluation.

## References

[1] Looker AC, Dallman PR, Carroll MD, Gunter EW, Johnson CL (1997). Prevalence of iron deficiency in the United States. JAMA.

[2] Nissenson AR, Wade S, Goodnough T, Knight K, Dubois RW (2005). Economic burden of anemia in an insured population. J Manag Care Pharm.

[3] Rockey DC (1999). Occult gastrointestinal bleeding. N Engl J Med.

[4] Tang SJ, Haber GB (2004). Capsule endoscopy in obscure gastrointestinal bleeding. Gastrointest Endosc Clin N Am.

[5] Furner M, Nagel R, Pinidiyapathirage J (2022). Video capsule endoscopy in patients with iron deficiency anaemia: experience at a regional Australian service. BMC Res Notes.

[6] Stone J, Grover K, Bernstein CN (2020). The use of capsule endoscopy for diagnosis of iron deficiency anemia: a retrospective analysis. J Clin Gastroenterol.

[7] Clere-Jehl R, Sauleau E, Ciuca S (2016). Outcome of endoscopy-negative iron deficiency anemia in patients above 65: a longitudinal multicenter cohort. Medicine (Baltimore).

[8] Lee JG, Galorport C, Yonge J, Enns RA (2020). Benefit of capsule endoscopy in the setting of iron deficiency anemia in patients above age 65. J Can Assoc Gastroenterol.

[9] Yung DE, Rondonotti E, Giannakou A (2017). Capsule endoscopy in young patients with iron deficiency anaemia and negative bidirectional gastrointestinal endoscopy. United European Gastroenterol J.

[10] Sidhu PS, McAlindon ME, Drew K, Sidhu R (2015). The utility of capsule endoscopy in patients under 50 years of age with recurrent iron deficiency anaemia: is the juice worth the squeeze?. Gastroenterol Res Pract.

[11] Holleran GE, Barry SA, Thornton OJ, Dobson MJ, McNamara DA (2013). The use of small bowel capsule endoscopy in iron deficiency anaemia: low impact on outcome in the medium term despite high diagnostic yield. Eur J Gastroenterol Hepatol.

[12] Goel RM, Patel KV, Borrow D, Anderson S (2014). Video capsule endoscopy for the investigation of the small bowel: primary care diagnostic technology update. Br J Gen Pract.

[13] Tong J, Svarta S, Ou G, Kwok R, Law J, Enns R (2012). Diagnostic yield of capsule endoscopy in the setting of iron deficiency anemia without evidence of gastrointestinal bleeding. Can J Gastroenterol.

[14] Riccioni ME, Urgesi R, Spada C, Cianci R, Pelecca G, Bizzotto A, Costamagna G (2010). Unexplained iron deficiency anaemia: is it worthwhile to perform capsule endoscopy?. Dig Liver Dis.

[15] Apostolopoulos P, Liatsos C, Gralnek IM (2006). The role of wireless capsule endoscopy in investigating unexplained iron deficiency anemia after negative endoscopic evaluation of the upper and lower gastrointestinal tract. Endoscopy.

[16] Koulaouzidis A, Yung DE, Lam JH, Smirnidis A, Douglas S, Plevris JN (2012). The use of small-bowel capsule endoscopy in iron-deficiency anemia alone; be aware of the young anemic patient. Scand J Gastroenterol.

[17] Koulaouzidis A, Rondonotti E, Giannakou A, Plevris JN (2012). Diagnostic yield of small-bowel capsule endoscopy in patients with iron-deficiency anemia: a systematic review. Gastrointest Endosc.

[18] Enns RA, Hookey L, Armstrong D (2017). Clinical practice guidelines for the use of video capsule endoscopy. Gastroenterology.

[19] Ko CW, Siddique SM, Patel A, Harris A, Sultan S, Altayar O, Falck-Ytter Y (2020). AGA clinical practice guidelines on the gastrointestinal evaluation of iron deficiency anemia. Gastroenterology.

[20] Snook J, Bhala N, Beales IL (2021). British Society of Gastroenterology guidelines for the management of iron deficiency anaemia in adults. Gut.

[21] Rockey DC, Altayar O, Falck-Ytter Y, Kalmaz D (2020). AGA technical review on gastrointestinal evaluation of iron deficiency anemia. Gastroenterology.

[22] Bytnar JA, Shriver CD, Zhu K (2021). Incidence rates of digestive cancers among U.S. military servicemen: comparison with the rates in the general U.S. population. PLoS One.

